# Tracking the implicit acquisition of nonadjacent transitional probabilities by ERPs

**DOI:** 10.3758/s13421-019-00949-x

**Published:** 2019-06-24

**Authors:** Andrea Kóbor, Kata Horváth, Zsófia Kardos, Ádám Takács, Karolina Janacsek, Valéria Csépe, Dezso Nemeth

**Affiliations:** 1 Brain Imaging Centre, Research Centre for Natural Sciences, Hungarian Academy of Sciences, Magyar tudósok körútja 2, Budapest, H–1117 Hungary; 2 Doctoral School of Psychology, ELTE Eötvös Loránd University, Izabella utca 46, Budapest, H–1064 Hungary; 3 Institute of Psychology, ELTE Eötvös Loránd University, Izabella utca 46, Budapest, H–1064 Hungary; 4 Brain, Memory and Language Research Group, Institute of Cognitive Neuroscience and Psychology, Research Centre for Natural Sciences, Hungarian Academy of Sciences, Magyar tudósok körútja 2, Budapest, H–1117 Hungary; 5 Department of Cognitive Science, Budapest University of Technology and Economics, Egry József utca 1, Budapest, H-1111 Hungary; 6 Lyon Neuroscience Research Center (CRNL), INSERM, CNRS, Université de Lyon, Centre Hospitalier Le Vinatier–Bâtiment 462–Neurocampus 95 Boulevard Pinel, 69675 Bron, Lyon France

**Keywords:** Implicit learning, Nonadjacent dependencies, P3, Predictive processes, Stimulus–response mapping

## Abstract

The implicit acquisition of complex probabilistic regularities has been found to be crucial in numerous automatized cognitive abilities, including language processing and associative learning. However, it has not been completely elucidated how the implicit extraction of second-order nonadjacent transitional probabilities is reflected by neurophysiological processes. Therefore, this study investigated the sensitivity of event-related brain potentials (ERPs) to these probabilistic regularities embedded in a sequence of visual stimuli without providing explicit information on the structure of the stimulus stream. Healthy young adults (*N* = 32) performed a four-choice RT task that included a sequential regularity between nonadjacent trials yielding a complex transitional probability structure. ERPs were measured relative to both stimulus and response onset. RTs indicated the rapid acquisition of the sequential regularity and the transitional probabilities. The acquisition process was also tracked by the stimulus-locked and response-locked P3 component: The P3 peak was larger for the sequence than for the random stimuli, while the late P3 was larger for less probable than for more probable short-range relations among the random stimuli. According to the RT and P3 effects, sensitivity to the sequential regularity is assumed to be supported by the initial sensitivity to the transitional probabilities. These results suggest that stimulus–response contingencies on the probabilistic regularities of the ongoing stimulus context are implicitly mapped and constantly revised. Overall, this study (1) highlights the role of predictive processes during implicit memory formation, and (2) delineates a potential to gain further insight into the dynamics of implicit acquisition processes.

The extraction and processing of probabilistic regularities underlying the environmental input is a powerful ability that contributes to the acquisition of automatic behaviors (Armstrong, Frost, & Christiansen, 2017; Aslin, 2017; Kaufman et al., 2010). Different types of probabilistic regularities can be simultaneously acquired from the same temporal sequence of sensory stimuli (Conway & Christiansen, 2001; Daltrozzo & Conway, 2014; Deocampo, King, & Conway, 2019; Siegelman, Bogaerts, Christiansen, & Frost, 2017; Thiessen, Kronstein, & Hufnagle, 2013). Regarding the different probabilistic regularities, it has been shown that humans are more proficient than nonhuman primates in extracting *nonadjacent* dependencies, referring to predictive relations or transitional probabilities between elements of a sequence that includes ordered stimuli interspersed with random ones (Malassis, Rey, & Fagot, 2018; Wilson et al., 2018). Thus, although the extraction of nonadjacent transitional probabilities appears to be an evolutionarily old process (Mueller, Milne, & Männel, 2018), it might also involve other human-specific cognitive computations (Malassis et al., 2018; Rey, Minier, Malassis, Bogaerts, & Fagot, 2018; Wilson et al., 2018) that might be reflected by particular neurophysiological processes (Maheu, Dehaene, & Meyniel, 2019).

A considerable amount of literature on the acquisition of probabilistic regularities has drawn conclusions from overt behavioral responses to the underlying neurophysiological processes, which might not be the most valid approach (Christiansen, 2018). Instead, the use of event-related brain potentials (ERPs) together with the analysis of behavioral responses can provide insight into the temporal resolution of various acquisition processes at the neurophysiological level. For instance, our previous findings indicate that acquisition processes related to certain types of probabilistic regularities can be distinguished at the level of ERPs, when the repeating regularity determining stimulus presentation is *explicitly* cued (Kóbor et al., 2018). However, although earlier neurophysiological research has contrasted the implicit (incidental) and explicit (intentional) aspects of learning temporal sequences (e.g., Batterink, Reber, & Paller, 2015; Daltrozzo & Conway, 2014; Ferdinand, Mecklinger, & Kray, 2008; Fu, Bin, Dienes, Fu, & Gao, 2013; Mueller et al., 2018; Verleger, Seitz, Yordanova, & Kolev, 2015), it has remained elusive to what degree ERPs are sensitive to the acquisition of second-order nonadjacent transitional probabilities without providing explicit information on the structure of the stimuli. Therefore, this study investigates the ERP correlates of implicitly acquiring these predictive relations occurring among visual stimuli in an active experimental task requiring key presses.

ERP research focusing on the implicit and explicit acquisition of probabilistic as well as deterministic regularities showed the sensitivity of the stimulus-related P3 component to predictive relations embedded in the stimulus sequence (Batterink, Reber, Neville, & Paller, 2015; Batterink, Reber, & Paller, 2015; Daltrozzo & Conway, 2014; Daltrozzo et al., 2017; Eimer, Goschke, Schlaghecken, & Stürmer, 1996; Ferdinand et al., 2008; Fogelson, 2015; Fu et al., 2013; Jongsma et al., 2006; Jongsma et al., 2013; Jost, Conway, Purdy, Walk, & Hendricks, 2015; Rose, Verleger, & Wascher, 2001; Rüsseler, Hennighausen, Münte, & Rösler, 2003; Rüsseler, Münte, & Wiswede, 2018; Rüsseler & Rösler, 2000; Schlaghecken, Stürmer, & Eimer, 2000; Stadler, Klimesch, Pouthas, & Ragot, 2006; Verleger, Seitz, et al., 2015). Traditionally, the P3 component, which is a large central or centroparietal positivity occurring between 300 ms and 600 ms after stimulus onset, has been found to indicate the conscious processing of action-related, task-relevant stimuli requiring decisions across a diverse range of experimental conditions (Kelly & O’Connell, 2015; Nieuwenhuis, Aston-Jones, & Cohen, 2005; O’Connell, Dockree, & Kelly, 2012; Polich, 2007; Ullsperger, Fischer, Nigbur, & Endrass, 2014). Moreover, the P3 has been linked to the processing of unexpected or surprising events (Mars et al., 2008; Sutton, Braren, Zubin, & John, 1965); and, accordingly, the P3 amplitude has appeared to be modulated by the subjective probability of the stimulus (e.g., Donchin, 1981; Donchin & Coles, 1988). Although the P3 is one of the most widely studied ERP components, its specific functional role in decision making is still under debate (Kelly & O’Connell, 2015; Kopp, 2007; Nieuwenhuis et al., 2005; Twomey, Murphy, Kelly, & O’Connell, 2015; Verleger, Jaśkowski, & Wascher, 2005).

Assumptions formulated in prior ERP research have been unspecific about which phase (e.g., the peak, the early, or the late phase) of, which parameter (e.g., amplitude or latency) of, and how (e.g., increase or decrease) the P3 component should be modulated by the acquisition of predictive relations. While some studies found larger P3 amplitudes for the less predictable events of the stimulus sequence than for the more predictable ones only if explicit knowledge about the underlying regularity was present during task solving (Eimer et al., 1996; Ferdinand et al., 2008; Fu et al., 2013; Rüsseler et al., 2003; Rüsseler & Rösler, 2000; Schlaghecken et al., 2000), others showed this modulation also in the case of implicitly acquired regularities (Jongsma et al., 2006; Jongsma et al., 2013; Mars et al., 2008; Rose et al., 2001), or both in the implicit and explicit experimental conditions (Batterink, Reber, Neville, et al., 2015; Batterink, Reber, & Paller, 2015). At the same time, in a handful of studies, larger P3 amplitudes were found for the more predictable events than for the less predictable ones in the explicit condition (Batterink, Reber, & Paller, 2015; Fogelson, Shah, Scabini, & Knight, 2009), in the implicit condition (Baldwin & Kutas, 1997; Daltrozzo et al., 2017; Jost et al., 2015; Rose et al., 2001; Rüsseler et al., 2018; Stadler et al., 2006), or both (Fogelson & Fernandez-del-Olmo, 2013). It seems that the P3 amplitude enhancement for the *more* rather than the less predictable events has usually been observed in highly structured tasks including short, repeating predictor–target sequences, in which the transitional probabilities between the predictors and the target have had to be extracted. Overall, P3 amplitude modulations in both directions could indicate the implicit sensitivity to or the implicit acquisition of predictable relations in a stimulus stream.

In the above-mentioned studies using choice-response tasks, only the stimulus-locked P3 component has been investigated, except for the study of Baldwin and Kutas (1997). It has been proposed, however, that if the P3 reflects the process of mapping a task-relevant stimulus onto an appropriate response, it should be related to both the stimulus and the response to the same degree (Folstein & van Petten, 2011; Stock, Steenbergen, Colzato, & Beste, 2016; Verleger et al., 2005). Therefore, the investigation of the stimulus-locked as well as the response-locked P3 would have been justified. In the earlier studies, the focus of the analysis was on the P3 peak measured in a narrow (e.g., Eimer et al., 1996; Ferdinand et al., 2008) or a wide time window (e.g., Fogelson et al., 2009), but often, a broad positive component was quantified and labeled as the P3 (e.g., Batterink, Reber, & Paller, 2015; Daltrozzo et al., 2017; Jost et al., 2015). Rarely, attempts have been made to differentiate components of the P3 complex (Verleger, Seitz, et al., 2015), and either the late (Rüsseler & Rösler, 2000) or the anticipatory/ascending (Batterink, Reber, & Paller, 2015; Stadler et al., 2006) phase of the P3 was analyzed beyond its peak. Overall, not only the question has remained whether amplitude modulations of the P3 could track the temporal trajectory of implicitly acquiring complex second-order nonadjacent transitional probabilities but also it has yet to be clarified which phase of the component in what direction would change as a reflection of this perceptual-cognitive process.

In this study, therefore, the processing-based or “online” effects of acquiring probabilistic regularities (Christiansen, 2018) were measured by a four-choice RT task that, unknown to participants, included a sequential regularity between nonadjacent trials (see Fig. 1). This task structure resulted in second-order transitional probabilities that, according to earlier behavioral (RT and accuracy) evidence (e.g., D. V. Howard et al., 2004; Janacsek, Ambrus, Paulus, Antal, & Nemeth, 2015; Nemeth et al., 2010; Takács et al., 2017; Tóth et al., 2017), can be acquired in an implicit manner, without consciously reporting the underlying regularity. Presumably, the continuous stream of observations over the course of the task could induce the gradual or rapid building up of predictions on the upcoming stimulus and thereby the formation of internal representations on the sequential regularity and the second-order transitional probability structure.

**Fig. 1:**
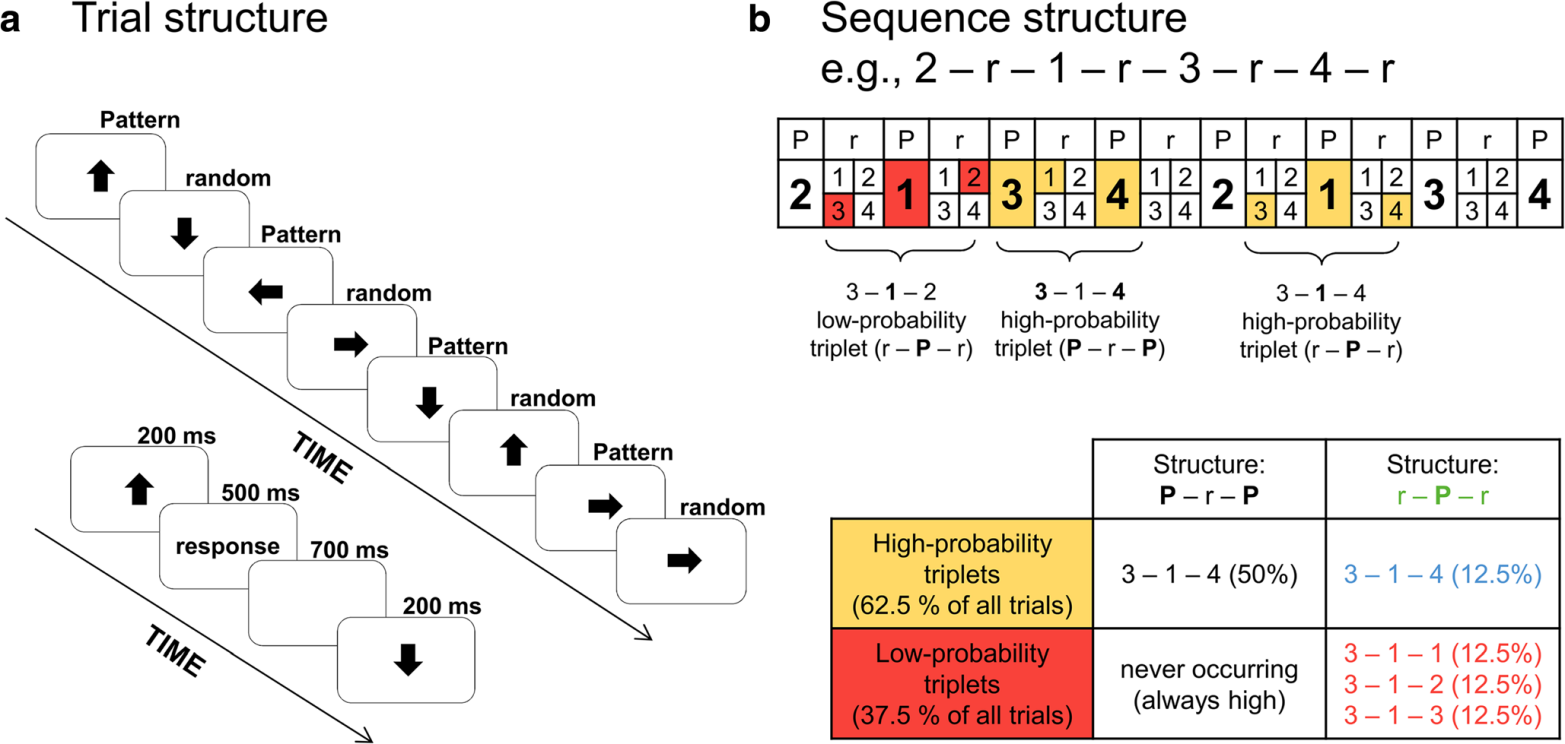
Design of the experiment. **a** In this version of the alternating serial reaction time (ASRT) task, an arrow stimulus appears at the center of the screen. The presentation of arrow stimuli follows an eight-element sequence, within which pattern (P) and random (r) elements alternate with one another. The timing of an arrow trial is presented below the trial structure. **b** In the alternating sequence structure, numbers denote the four spatial directions (1 = left, 2 = up, 3 = down, 4 = right) of the arrows. The alternating sequence makes some runs of three consecutive trials (triplets) more probable than others. High-probability triplets are denoted with gold shading, and low-probability triplets are denoted with coral shading. Among high-probability triplets, pattern (with black font in the lower table) and random (with blue font in the lower table) high-probability triplets are distinguished. In the random triplet category (r–P–r structure, see the green font in the lower table), random low-probability triplets are also distinguished (with coral font in the lower table). (Color figure online)

Accordingly, if changes of the P3 component tracked the acquisition of nonadjacent transitional probabilities, we assume that its amplitude would be larger for the less probable than for the more probable stimuli of the sequence (cf. Batterink, Reber, Neville, et al., 2015; Jongsma et al., 2006; Maheu et al., 2019; Mars et al., 2008). This hypothesis holds for the peak as well as the late phase of the P3. However, it is presumable that sensitivity to the sequential regularity would be observed first and sensitivity to the transitional probabilities would be observed later in time, reflected by the peak and the late phase of the P3, respectively. Namely, the P3 peak amplitude would be larger for those stimuli that do not follow the sequential regularity (random stimuli) than for the regular ones, while the late P3 amplitude would be larger for the less probable than for the more probable short-range relations occurring because of the second-order transitional probability structure. In addition, if the P3 were related to stimulus–response mapping, the acquisition of both regularities should be reflected in stimulus-locked and response-locked averages to a similar extent; therefore, the formulated assumptions should be relevant also for the response-locked P3. As previous studies predominantly focused on the amplitude of the P3 instead of the latency, in this study, we do not analyze latencies. Based on earlier behavioral studies, RTs are assumed to become increasingly faster to regular than to random stimuli and to more probable than to less probable short-range relations over the task.

## Method

### Participants

Thirty-two healthy young adults (24 females) between the ages of 19 and 26 years (*M* = 21.3, *SD* = 1.7) took part in the experiment. They were undergraduate students from Budapest, Hungary (years of education: *M* = 14.5, *SD* = 1.6). Handedness was assessed with the Edinburgh Handedness Inventory revised version (Dragovic, 2004a, 2004b; Oldfield, 1971), according to which the mean Laterality Quotient for right-handers (*n* = 27) was 85.2 (*SD* = 13.1), for left-handers (*n* = 4), it was −70.3 (*SD* = 21.3), and for the mixed-handed participant (*n* = 1), it was −37.5 (−100 means complete left-handedness, 100 means complete right-handedness). Participants had normal or corrected-to-normal vision, and according to the pre-defined inclusion criteria, none of them reported a history of any neurological and/or psychiatric condition, and none of them was taking any psychoactive medication. They performed in the normal range on standard neuropsychological tests (Wisconsin Card Sorting Task [perseverative error percentage]: *M* = 11.81, *SD* = 2.75; digit-span task [mean short-term memory span; possible range: 3–9]: *M* = 6.38, *SD* = 1.54; counting-span task [mean working memory span; possible range: 2–6]: *M* = 3.21, *SD* = 0.54; verbal fluency task [total number of correct items in phonemic and semantic subtasks]: *M* = 54.84, *SD* = 10.33; go/no-go task [discriminability index: hit rate minus false alarm rate]: *M* = .66, *SD* = .20). All participants provided written informed consent before enrollment and received payment (ca. 12 euros) or course credit for taking part in the experiment. The study was approved by the United Ethical Review Committee for Research in Psychology (EPKEB) in Hungary and was conducted in accordance with the Declaration of Helsinki.

### Stimuli, task, and procedure

Implicit acquisition of probabilistic regularities was measured by a version of the alternating serial reaction time (ASRT) task (Nemeth et al., 2010; Takács et al., 2018), which was optimized for EEG/ERP measurement (Horváth, Kardos, et al., 2019; Kóbor et al., 2018). In this task, a black arrow stimulus appears at the center of the screen. Participants are instructed to press one of the four response keys of a Cedrus RB-530 response pad (Cedrus Corporation, San Pedro, CA) as quickly and accurately as possible. The four response keys correspond to the spatial directions of the arrow stimuli (left [left thumb], up [left index finger], down [right thumb], or right [right index finger]). Arrow images pointing up and down had a width of 1.06° and height of 1.61° in visual angle, while those pointing left and right had a width of 1.61° and a height of 1.06° in the present experiment.

Unbeknownst to the participants, arrow stimuli are presented according to an eight-element sequence, within which predetermined/pattern (P) and random (r) elements alternate with one another (see Fig. 1a). For instance, 2–r–1–r–3–r–4–r is one of the sequences, where numbers denote the four predetermined spatial directions [1 = left, 2 = up, 3 = down, 4 = right] of the arrows and *r*s denote the randomly chosen directions out of the four possible ones (see Fig. 1b). There are 24 permutations of the four spatial directions that could be applied as the sequence; however, because of the continuous presentation of the stimuli, there are only six unique permutations. In this study, one of these six unique permutations was selected for each participant in a pseudorandom manner (see also J. H. Howard & Howard, 1997; Kóbor et al., 2018; Nemeth et al., 2010).

The alternating sequence yields a probability structure in which some chunks of three successive trials—hereafter referred to as *triplets—*occur more frequently than others. In the case of the 2–r–1–r–3–r–4–r sequence, 2–X–1, 1–X–3, 3–X–4, and 4–X–2 triplets (X denotes the middle trial of the triplet) occur frequently since these triplets could have P–r–P or r–P–r structure. Meanwhile, for instance, 1–X–2 and 4–X–3 triplets occur less frequently since they could only have a r–P–r structure (see Fig. 1b). The former triplets are referred to as high-probability triplets while the latter ones are referred to as low-probability triplets (e.g., Nemeth & Janacsek, 2011; Nemeth, Janacsek, Polner, & Kovacs, 2013). Construction of triplets could be considered as a method for identifying a hidden probability structure of the ASRT task, with the help of which predetermined and random elements of the alternating sequence can be further categorized based on probability. These triplet types describe not only the distributional (the frequency of the triplet) but also the second-order transitional probabilities embedded in the task. Namely, the final trial of a high-probability triplet is a probable (predictable) continuation for the first trial of the triplet while the final trial of a low-probability triplet is a less probable continuation. For instance, if the first trial of a triplet is spatial direction 3, it is more likely (with 62.5% probability) to be followed by spatial direction 4 as the third trial than either spatial direction 1, 2, or 3 (with 12.5% probability each; see Fig. 1b). Each trial (arrow stimulus) is categorized as either the third trial of a high-probability or a low-probability triplet. Accordingly, the construction of triplets is applied as a moving window throughout the entire stimuli set: The third trial of a triplet is also the second trial of the following triplet, and so on; thus, all stimuli are categorized this way (Kóbor, Janacsek, Takács, & Nemeth, 2017; Kóbor et al., 2018; Szegedi-Hallgató et al., 2017). There are 64 possible triplets in the task: 16 of them are high-probability triplets and 48 are low-probability ones. With respect to the unique triplets, high-probability triplets occur five times more often than low-probability ones.

Because of the alternating sequence, random trials that are the 50% of all trials appear either with high or low probability, while the pattern trials that are the other 50% of all trials always appear with high probability. Overall, the combination of the sequential and probability properties yields three possible trial [triplet] types: pattern high-probability, random high-probability, and random low-probability triplets (occurring with an overall probability of 50%, 12.5%, and 37.5%, respectively; see Fig. 1b). In relation to the task structure, note that low-probability triplets are always random, the random triplet category consists of random high-probability and low-probability triplets, and that the terms “pattern triplets” and “pattern high-probability triplets” are interchangeable.

The structure of the task and the structure and timing of an experimental trial were similar to that reported in the Kóbor et al. (2018) paper. An experimental trial started with the presentation of the arrow stimulus at the center of the screen for 200 ms, then a blank screen was displayed until participants gave a behavioral response (key press) but no longer than 500 ms. Following the correct/incorrect response or the duration of 500 ms if no response occurred (missing response), a blank screen was presented again for a fixed delay of 700 ms (response-to-stimulus interval = RSI) before the start of the next trial (see Fig. 1a). Participants could also respond during stimulus presentation; in this case, the stimulus disappeared from the screen after response onset, and only the RSI blank screen was presented. In incorrectly responded trials, a blank screen was presented for 500 ms after response onset, and then an “X” appeared at the center of the screen for another 500 ms as a feedback on the incorrect response. This event was followed by the 700-ms-long RSI. If no response occurred during stimulus presentation and the 500 ms response window, a “!” was displayed for 500 ms, followed by the RSI. After an incorrect or missing response, although participants could provide further behavioral responses, this did not influence the presentation and timing of the next trial. Similarly, participants could proceed with the trial without providing the correct response. Importantly, only correctly responded trials were analyzed in the present study.

One block of the ASRT task contained 85 trials (stimuli). In each block, the eight-element sequence repeated 10 times after five warm-up trials consisting only of random stimuli. After each block, participants received feedback (lasting for 4,000 ms) about their mean reaction time and accuracy in the given block, then they could have a short rest before starting the next block. Altogether 30 blocks were completed (2,550 trials in total). After completing the ASRT task, to test the implicitness of the acquired knowledge about the probabilistic regularities, the inclusion–exclusion task (Destrebecqz & Cleeremans, 2001; Destrebecqz et al., 2005; Fu, Dienes, & Fu, 2010a, 2010b; Horváth, Török, Pesthy, Nemeth, & Janacsek, 2019; Kóbor et al., 2017) was administered. This task is based on the process dissociation procedure (Jacoby, 1991). In the first, inclusion condition of the task, participants are required to produce a sequence of key presses that follows the order in which the arrow stimuli appeared in the ASRT task. Second, in the exclusion condition, participants are required to produce a sequence of key presses according to a new order that they did not observe during the task. Both conditions of the inclusion–exclusion task consist of four runs, each run finishes after 24 key presses, and participants use the same response keys as in the ASRT task. Since the produced sequences of stimuli include both pattern and random elements, this task could measure whether the participants’ knowledge about the different triplet types is consciously accessible. Therefore, and according to the above-referred studies, successful performance in the inclusion condition can be achieved using both implicit and explicit knowledge about the probabilistic regularities. However, in the exclusion condition, consciously accessible knowledge is required about these regularities to inhibit the original order of stimuli as appeared in the task and thereby to produce a different sequence of key presses. In this task condition, failure to inhibit the original sequence indicates implicit knowledge. Hence, to test whether participants gained consciously accessible knowledge about the probabilistic regularities, first, the percentage of high-probability triplets (pattern and random high-probability triplets) that participants produced during the inclusion and exclusion conditions, respectively, was calculated. Then, it was tested whether participants produced more high-probability triplets than it would have been expected by chance in each of the conditions, and whether the percentage of high-probability triplets differed between the conditions (see also Horváth, Török, et al., 2019; Kóbor et al., 2017).

The experimental procedure lasted about 2.5 hours, including the application and removal of the electrode cap. The ASRT task and the inclusion–exclusion task were written in and controlled by the Presentation software (Version 18.1, Neurobehavioral Systems). Stimuli were displayed on a 21-in. LCD screen at a viewing distance of 125 cm. Neuropsychological tests (see Participants section) were administered a few days before the EEG experiment during a 1-hour-long session.

### EEG recording and analysis

The continuous EEG activity was recorded in an electrically shielded, acoustically attenuated, and dimly lit room using the actiCAP active electrode system with BrainAmp Standard amplifier and BrainVision Recorder 1.2 software (BrainProducts GmbH, Munich, Germany). The 64 sensors consisting of Ag/AgCl electrodes integrated with active circuits were mounted in an elastic cap and placed according to the 10% equidistant system. The FCz electrode was used as reference and the Fpz electrode was used as ground. The sampling rate was 1000 Hz; and during recording, the impedance of the electrodes was kept below 10 kΩ.

The continuous EEG data were analyzed off-line using the BrainVision Analyzer 2.0 software (BrainProducts GmbH). The preprocessing steps described below followed those presented in the Kóbor et al. (2018) paper. First, after visual screening for major deflections, if necessary, bad electrodes were replaced by spline interpolation: Electrodes between zero and two per participant (*M* = 0.28, *SD* = 0.63) were interpolated. Second, the EEG data were band-pass filtered within 0.5–30 Hz (48 dB/oct) and notch filtered at 50 Hz to remove additional electrical noise. Third, horizontal and vertical eye-movement artifacts and heartbeats were corrected with independent component analysis (Delorme, Sejnowski, & Makeig, 2007): Components between one and three per participant (*M* = 2.19, *SD* = 0.54) were rejected, then, the channel-based EEG data were recomposed. Fourth, EEG data were rereferenced to the average activity of all electrodes. Fifth, the continuous EEG was segmented in two steps as follows. To track the temporal trajectory of acquisition, the data were cut into six, equally long time bins (epochs), each containing five consecutive blocks of the ASRT task. Next, for stimulus-locked averages, within each epoch, segments were extracted from −200 ms to 600 ms relative to stimulus onset, separately for pattern, random high-probability, random low-probability, and random (including random high-probability and low-probability) triplets [trials]. For response-locked averages, within each epoch, segments were extracted from −700 ms to 700 ms relative to response onset, separately for the same triplet types. Only correctly responded trials with an RT greater than zero ms were included in this step of the segmentation. Altogether 24 (four triplet types × six epochs) segment types were created for both stimulus-locked and response-locked averages. Note that following the standard data analysis protocol established in previous studies using the ASRT task (e.g., J. H. Howard & Howard, 1997; Kóbor et al., 2017; Nemeth, Janacsek, Polner, et al., 2013; Song, Howard, & Howard, 2007; Virag et al., 2015), two types of low-probability triplets—repetitions (e.g., 1–1–1, 4–4–4) and trills (e.g., 1–2–1, 2–4–2)—were eliminated from the behavioral and ERP analyses, because preexisting response tendencies have often been shown to them (D. V. Howard et al., 2004). Therefore, the low-probability triplet category consisted of low-probability triplets without trills and repetitions.

Following segmentation, to remove artifacts still present in the data after ICA corrections, an automatic artifact rejection algorithm implemented in the BrainVision Analyzer software was applied, which rejected segments where the activity exceeded ±100 μV at any of the electrode sites. The mean percentage of removed stimulus-locked segments across the three basic triplet types (pattern, random high-probability, and low-probability triplets) was 0.34% (*SD* = 1.31%, range: 0%–18.18%). The mean percentage of removed response-locked segments across the same triplet types was 0.89% (*SD* = 2.97%, range: 0%–34.09%). As the percentage of removed segments was below 35% for each triplet type, all participants’ data were included in further analysis. Accordingly, the mean numbers and ranges of retained segments for stimulus-locked data were 181.2 (range: 141–194) for pattern triplets, 45.5 (range: 27–59) for random high-probability triplets, and 89.5 (range: 66–106) for low-probability triplets. For response-locked data, these numbers were 180.3 (range: 123–194) for pattern triplets, 45.2 (range: 27–59) for random high-probability triplets, and 89.0 (range: 66–106) for low-probability triplets. The retained stimulus-locked segments were baseline corrected based on the mean activity from −200 ms to 0 ms (prestimulus baseline). The retained response-locked segments were baseline corrected based on the mean activity from 500 ms to 700 ms, which was the last 200-ms-long interval before the next stimulus onset (i.e., it was the end of the RSI interval, which corresponded to the prestimulus baseline used for stimulus-locked averaging). Finally, these segments were averaged for all four (pattern, random high-probability, random low-probability, and random) triplet types in each of the six epochs.

Grand average ERP waveforms calculated separately for each triplet type in each epoch as well as averaged for the entire acquisition phase across all epochs were visually inspected to determine the latency range where the P3 component might vary as a function of triplet types. First, the peak of the P3 was quantified as the mean amplitude between 280 ms and 380 ms after stimulus onset in the stimulus-locked averages, because the grand average peak for all triplet types at the electrode CPz (where this ERP component showed maximum amplitude) was at approx. 330 ms, around which a ±50-ms latency range was determined. Similarly, in the response-locked averages, the peak of the P3 was quantified as the mean amplitude within 50 ms before to 50 ms after response onset where the grand average peak appeared at the electrode CPz. Second, the late descending flank of the P3 (henceforth referred to as late P3) was quantified in the remaining interval of the segment in stimulus-locked averages (i.e., between 380 ms and 600 ms). The late P3 in response-locked averages was quantified between 50 ms and 250 ms after response onset, since a negative deflection started at approx. 250 ms at the electrode CPz. Based on the variations of the grand average ERPs and the observed and previously reported topographical distribution of the P3 component, a centroparietal (CP) electrode pool was defined by calculating the average activity of the electrodes CP1, CPz, CP2, P1, Pz, and P2. The P3 peak and the late P3 in both stimulus-locked and response-locked averages was quantified over this CP pool.

### Data analysis

We measured the acquisition of the probability structure of the ASRT task in two steps. First, to quantify whether participants acquired that there was an oscillation of a repeating sequence in which predetermined/pattern and random stimuli alternated, we contrasted pattern and random triplets. Second, to quantify whether participants acquired the second-order nonadjacent transitional probabilities, we contrasted all three triplet types (pattern, random high-probability, and random low-probability triplets) to one another. In both cases, we focused on how RTs and the P3 amplitude change over the course of the task. In addition, we followed the change in these indices related particularly to the processing of random high-probability triplets. Sensitivity to these triplets overarches the sensitivity to both regularities (i.e., the pattern vs. random alternation and the nonadjacent transitional probabilities), because they are “accidentally regular” random triplets. If the processing of random high-probability triplets lays in-between the processing of pattern triplets and that of the random low-probability ones, it would mean that both the sequential regularity (P–r–P–r information) and the regularity of transitional probabilities (information on the high-probability and low-probability chunks) would be acquired. Accumulating behavioral evidence indicates that participants respond increasingly faster to high-probability triplets compared with low-probability ones as the ASRT task progresses (e.g., Janacsek et al., 2015; Kóbor et al., 2017; Nemeth et al., 2010; Nemeth, Janacsek, Polner, et al., 2013; Takács et al., 2017; Tóth et al., 2017). However, contrasting only high-probability versus low-probability triplets obscures how exactly the probability of short-range relations has been acquired and/or how this knowledge might have been transferred from the pattern high-probability (P–r–P) triplets to the case of random stimuli. Independent of triplet types, general skill improvements (faster RTs) could also be measured in the ASRT task, which reflect more efficient visuomotor and motor-motor coordination due to practice (Hallgató, Győri-Dani, Pekár, Janacsek, & Nemeth, 2013).

Similarly to the segmentation of the EEG data, five-block-long segments of the behavioral data were grouped into larger time bins (epochs). Accordingly, we altogether analyzed six epochs of the ASRT task, which are labeled consecutively in this paper (1, 2, etc.). Regarding the behavioral data, for each participant and epoch, separately for pattern, random high-probability, random low-probability, and random triplets (random high-probability and low-probability triplets together), median RT was calculated only for correct responses with an RT greater than zero ms. For the sake of completeness, we report the mean accuracy of responses for each triplet type and epoch in Table 1. However, because of the accuracy of responding has been influenced by the feedback given to participants after each block, and overall accuracy has usually been high with relatively low variability in samples of healthy young adults performing the ASRT task (J. H. Howard & Howard, 1997; Janacsek et al., 2015; Nemeth et al., 2010; Romano, Howard, & Howard, 2010), we focus on the RT analysis and do not analyze accuracy data in details here. In addition, since RTs and ERPs are calculated only for correctly responded trials, accuracy results are not assumed to match RT and ERP results, and, therefore, are not considered (for the same approach, see Kóbor et al., 2018).

**Table 1:** Mean percentage (%) and standard deviation of response accuracy split by triplet type and epoch

	Pattern	Random High	Random Low	Random
	*M* (*SD*)	*M* (*SD*)	*M* (*SD*)	*M* (*SD*)
Epoch_1_	93.4 (3.5)	92.5 (3.7)	92.6 (4.0)	92.6 (3.6)
Epoch_2_	93.2 (4.0)	93.3 (4.7)	91.4 (4.5)	92.2 (3.9)
Epoch_3_	93.5 (3.8)	95.3 (3.6)	92.2 (5.6)	93.3 (4.5)
Epoch_4_	93.0 (4.8)	94.2 (4.7)	91.6 (4.4)	92.4 (3.8)
Epoch_5_	93.1 (3.1)	93.9 (3.8)	91.8 (4.5)	92.5 (3.8)
Epoch_6_	93.0 (5.0)	94.5 (4.4)	91.5 (5.4)	92.5 (4.8)

At the behavioral level, the acquisition of the sequential regularity was quantified with a two-way repeated-measures analysis of variance (ANOVA), with Type (pattern vs. random triplet) and Epoch (1–6) as within-subjects factors on the RTs. The acquisition of second-order transitional probabilities was quantified with another Type × Epoch ANOVA, where the Type factor contrasted pattern high-probability, random high-probability, and random low-probability triplets. At the ERP level, the acquisition of the sequential regularity was quantified with a three-way repeated-measures ANOVA, with Locking (stimulus-locked vs. response-locked averages), Type (pattern vs. random triplet), and Epoch (1–6) as within-subjects factors on the P3 peak and the late P3. Again, on the same dependent measures, the acquisition of second-order transitional probabilities was quantified with another Locking × Type × Epoch ANOVA, where the Type factor contrasted pattern high-probability, random high-probability, and random low-probability triplets. In all ANOVAs, the Greenhouse–Geisser epsilon (ε) correction (Greenhouse & Geisser, 1959) was used when necessary. Original *df* values and corrected (if applicable) *p* values are reported together with partial eta-squared (η_p_^2^) as the measure of effect size. When the significant main effects and interactions were followed up, LSD (least significant difference) tests for pair-wise comparisons were used to control for Type I error. If no significant Type × Epoch interaction emerged from the overall ANOVA contrasting pattern high-probability, random high-probability, and random low-probability triplets, the temporal trajectory of the three triplet types was quantified separately with one-way repeated-measures ANOVAs, with Epoch as a within-subjects factor, followed up with LSD tests.

## Results

Results from ANOVAs performed on behavioral and ERP data are presented in Table 2.

**Table 2:** Summary of results from ANOVAs performed on behavioral and ERP data.

Effect	Statistics	RT		P3 peak		Late P3	
		Pattern vs. Random	Overall	Pattern vs. Random	Overall	Pattern vs. Random	Overall
Type	*F*	25.32	61.45	21.87	5.40	2.03	17.37
	*p*	**<.001**	**<.001**	**<.001**	**.017**	.164	**<.001**
	η_p_^2^	.450	.665	.414	.148	.062	.359
Epoch	*F*	4.53	3.37	1.16	1.40	5.74	5.90
	*p*	**.004**	**.016**	.328	.246	**.002**	**.002**
	η_p_^2^	.128	.098	.036	.043	.156	.160
Type × Epoch	*F*	1.98	1.90	1.04	1.76	0.61	2.56
	*p*	.085	.076	.398	.108	.690	**.021**
	η_p_^2^	.060	.058	.032	.054	.019	.076
Locking	*F*	–	–	3.33	3.47	2.18	1.91
	*p*	–	–	.078	.072	.150	.177
	η_p_^2^	–	–	.097	.101	.066	.058
Locking × Type	*F*	–	–	0.54	0.75	0.79	0.79
	*p*	–	–	.469	.477	.381	.457
	η_p_^2^	–	–	.017	.024	.025	.025
Locking × Epoch	*F*	–	–	0.57	0.52	1.72	1.68
	*p*	–	–	.641	.696	.155	.160
	η_p_^2^	–	–	.018	.016	.053	.051
Locking × Type × Epoch	*F*	–	–	1.71	1.05	1.37	0.87
	*p*	–	–	.136	.405	.237	.562
	η_p_^2^	–	–	.052	.033	.042	.027

*p* values below .050 are **boldfaced**

### Behavioral results

**Fig. 2:**
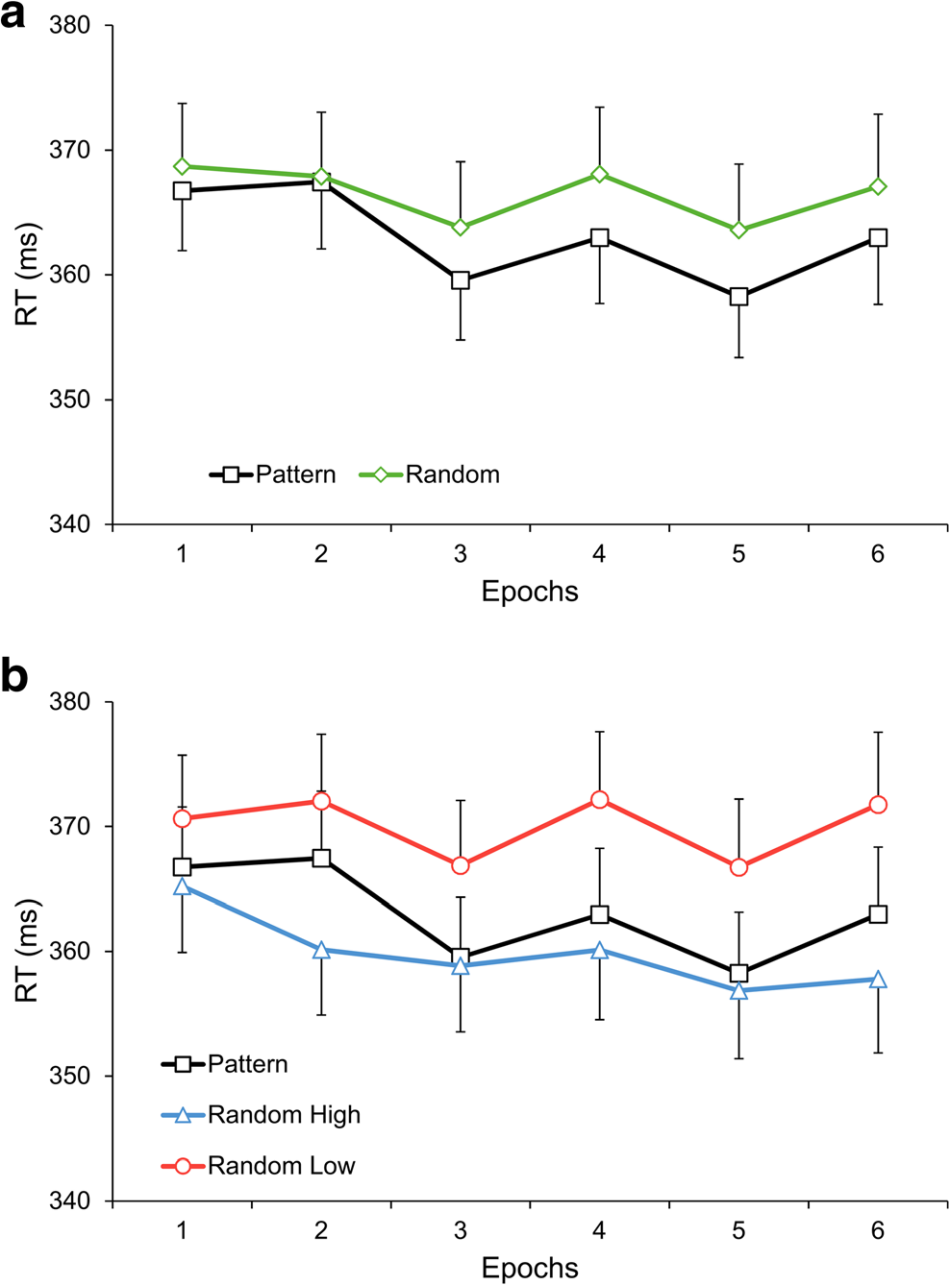
Behavioral results suggesting sensitivity to the probability structure of the task. **a** Group-average RTs for correct responses as a function of epoch (1–6) and triplet type (pattern vs. random triplets) showing sensitivity to the sequential regularity. **b** Group-average RTs for correct responses as a function of epoch (1–6) and triplet type (pattern and random high-probability triplets and random low-probability triplets) showing sensitivity to the second-order transitional probabilities. Error bars denote standard error of mean

The Type (pattern vs. random triplets) × Epoch (1–6) ANOVA on the RTs revealed sensitivity to the sequential regularity and general skill improvements (significant main effects of Type, *F*(1, 31) = 25.32, *p* < .001, η_p_^2^ = .450, and Epoch, *F*(5, 155) = 4.53, ε = .676, *p* = .004, η_p_^2^ = .128). Participants were faster on pattern than on random triplets (363 ms vs. 367 ms), but this difference did not reliably change with practice (the Type × Epoch interaction was only a tendency, *F*(5, 155) = 1.98, *p* = .085, η_p_^2^ = .060; see Fig. 2a). To detail general skill improvements, RTs were significantly faster in epoch_3_ (362 ms) than in epochs_1,2,4_ (all *p*s ≤ .008), and they were faster in epoch_5_ (361 ms) than in epochs_1,2,4,6_ (all *p*s ≤ .023).

The Type × Epoch ANOVA contrasting pattern high-probability, random high-probability, and random low-probability triplets revealed sensitivity to the second-order transitional probabilities as well as general skill improvements (significant main effects of Type, *F*(2, 62) = 61.45, *p* < .001, η_p_^2^ = .665, and Epoch, *F*(5, 155) = 3.37, ε = .698, *p* = .016, η_p_^2^ = .098. Participants were the fastest on random high-probability triplets (random vs. pattern high-probability triplets: 360 ms vs. 363 ms, *p* = .001; random high-probability vs. low-probability triplets: 360 ms vs. 370 ms, *p* < .001), and they were also significantly faster on pattern high-probability than on random low-probability triplets (363 ms vs. 370 ms, *p* < .001). However, the difference in RTs across triplet types did not reliably change with practice (the Type × Epoch interaction was only a tendency, *F*(10, 310) = 1.90, ε = .654, *p* = .076, η_p_^2^ = .058; see Fig. 2b). To detail general skill improvements, RTs, again, were significantly faster in epoch_3_ (362 ms) than in epochs_1,2,4_ (all *p*s ≤ .036), and they were faster in epoch_5_ (361 ms) than in epochs_1,2,4_ (all *p*s ≤ .034). Treating the three triplet types separately, a one-way ANOVA, with Epoch as a within-subjects factor, did not yield a significant effect on random high-probability triplets, *F*(5, 155) = 1.64, *p* = .152, η_p_^2^ = .050. Similar one-way ANOVAs indicated a significant change of RTs as the task progressed on pattern high-probability triplets, *F*(5, 155) = 7.09, ε = .745, *p* < .001, η_p_^2^ = .186, and a tendency on random low-probability triplets, *F*(5, 155) = 2.17, *p* = .061, η_p_^2^ = .065. RTs on pattern high-probability triplets were faster in epoch_3_ (360 ms) than in epochs_1,2_ (all *p*s < .001), they were faster in epoch_5_ (358 ms) than in epochs_1,2,4,6_ (all *p*s ≤ .002), and they were slower in epoch_2_ (367 ms) than in epochs_4,6_ (all *p*s ≤ .032).

Regarding the inclusion–exclusion task, during analysis, data of two participants from the inclusion condition and data of one participant from the exclusion condition were excluded because of not following the task instructions. In the inclusion condition, participants produced 6.52% more high-probability triplets than it would have been expected by chance (chance level: 25%), *t*(29) = 4.34, *p* < .001. In the exclusion condition, participants also produced 3.14% more high-probability triplets than the chance level, *t*(30) = 2.46, *p* = .020, suggesting that they could not consciously inhibit the acquired knowledge about the probabilistic regularities. The production of high-probability triplets was greater in the inclusion than in the exclusion condition, *t*(28) = −2.93, *p* = .007.

### P3 results

**Fig. 3:**
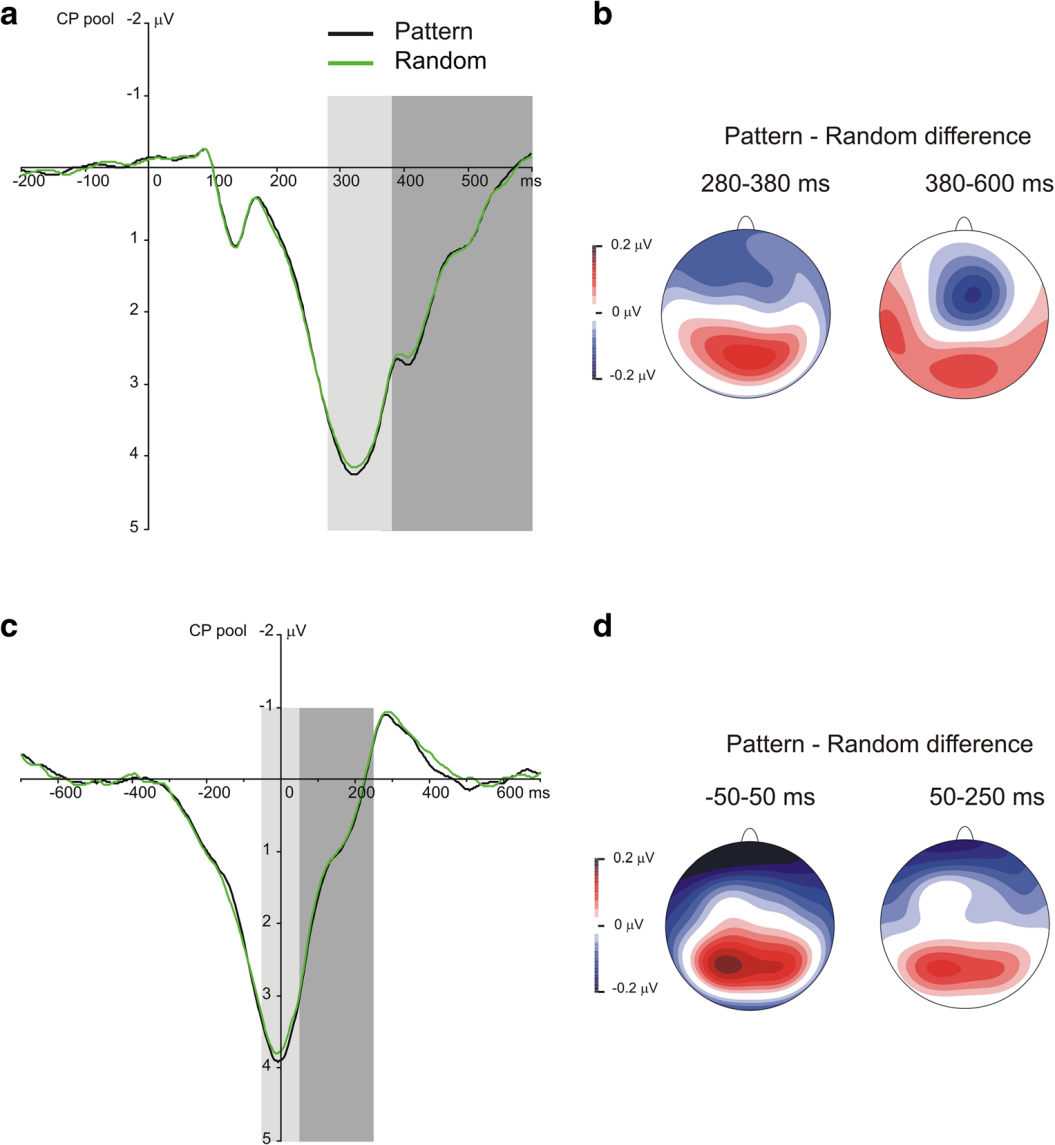
Stimulus-locked and response-locked P3 results suggesting sensitivity to the sequential regularity. **a** Grand average stimulus-locked ERP waveforms over the centroparietal electrode pool are presented, displaying the P3 component for random and pattern triplets, averaged for all epochs. The light-gray shaded area indicates the time window in which the P3 peak was quantified (280–380 ms); the dark-gray shaded area indicates the time window in which the late P3 was quantified (380–600 ms). Zero ms indicates stimulus onset. **b** The scalp topography (amplitude distribution) of stimulus-locked ERP differences for pattern minus random triplets in both time windows. **c** Grand average response-locked ERP waveforms over the centroparietal electrode pool are presented, displaying the P3 component for random and pattern triplets, averaged for all epochs. The light-gray shaded area indicates the time window in which the P3 peak was quantified (−50–50 ms); the dark-gray shaded area indicates the time window in which the late P3 was quantified (50–250 ms). Zero ms indicates response onset. **d** The scalp topography (amplitude distribution) of response-locked ERP differences for pattern minus random triplets in both time windows

**Fig. 4:**
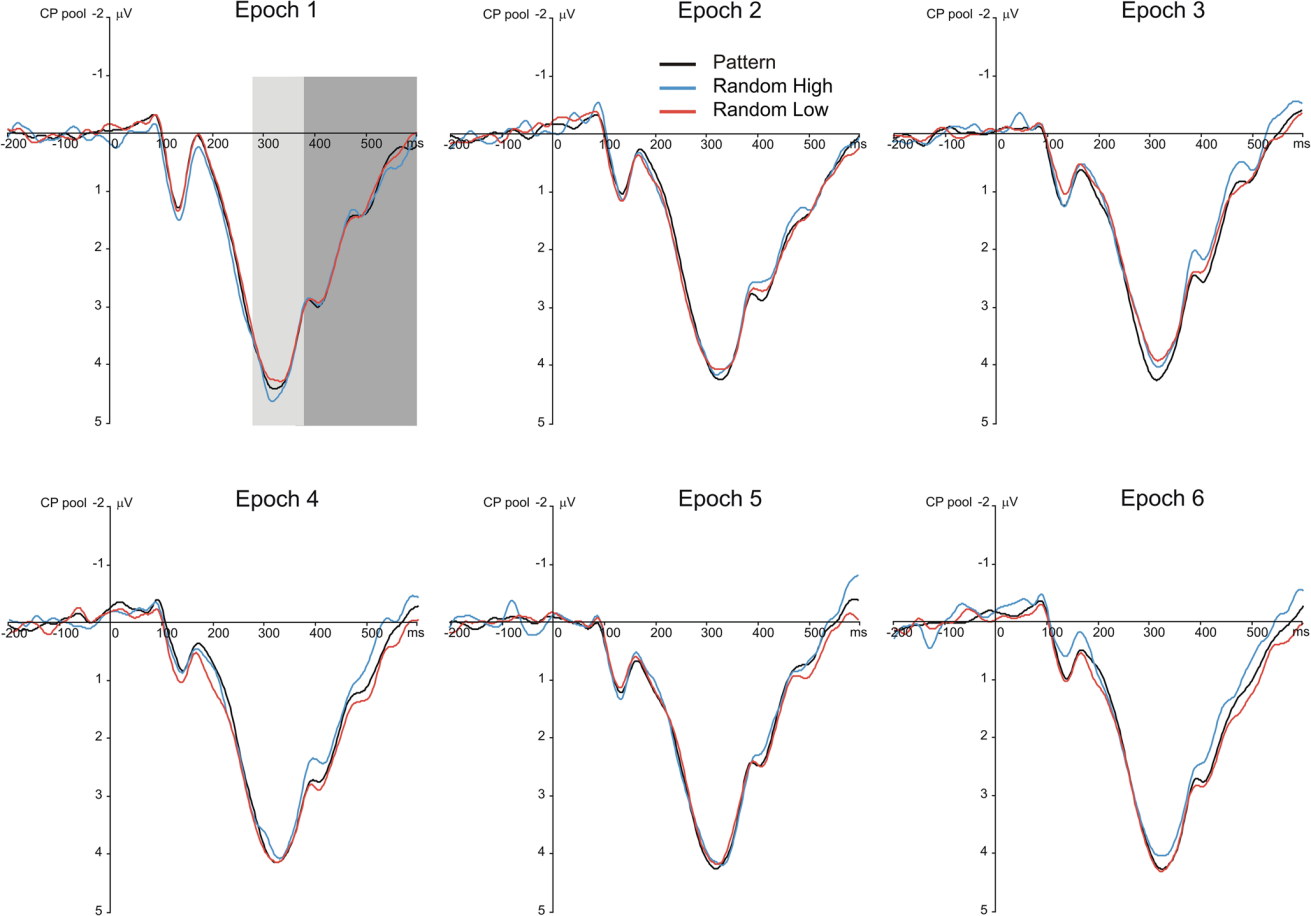
Stimulus-locked P3 results suggesting sensitivity to the probability structure of the task. Grand average stimulus-locked ERP waveforms over the centroparietal electrode pool are presented, displaying the P3 component for each epoch (1–6) and triplet type (pattern and random high-probability triplets and random low-probability triplets). The light-gray shaded area indicates the time window in which the P3 peak was quantified (280–380 ms); the dark-gray shaded area indicates the time window in which the late P3 was quantified (380–600 ms). Zero ms indicates stimulus onset

**Fig. 5:**
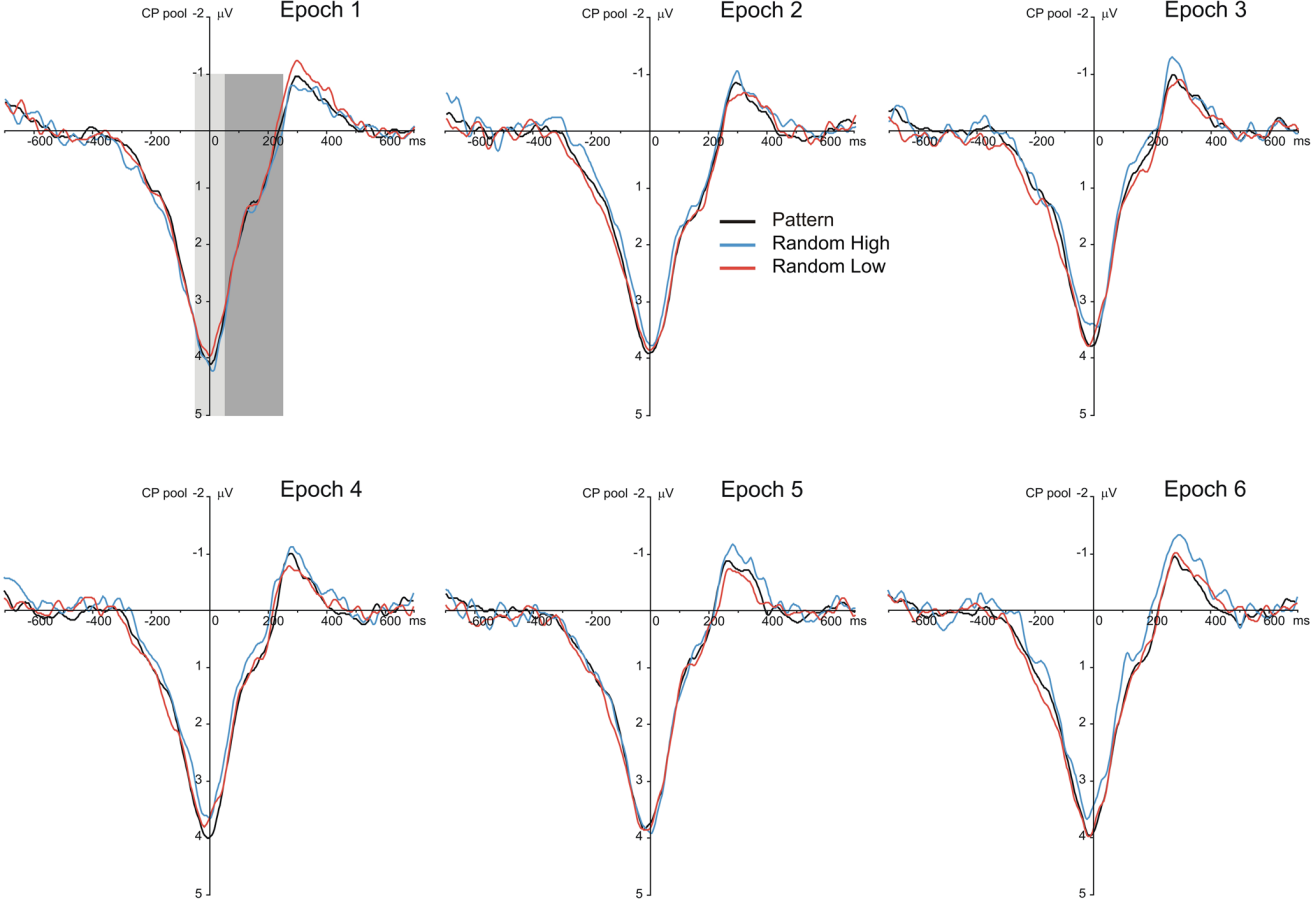
Response-locked P3 results suggesting sensitivity to the probability structure of the task. Grand average response-locked ERP waveforms over the centroparietal electrode pool are presented, displaying the P3 component for each epoch (1–6) and triplet type (pattern and random high-probability triplets and random low-probability triplets). The light-gray shaded area indicates the time window in which the P3 peak was quantified (−50–50 ms); the dark-gray shaded area indicates the time window in which the late P3 was quantified (50–250 ms). Zero ms indicates response onset

Stimulus-locked and response-locked grand average ERP waveforms split by triplet type and epoch over the centroparietal electrode pool are presented in Figs. 3, 4, and 5.

#### P3 peak

The Locking (stimulus-locked vs. response-locked averages) × Type (pattern vs. random triplets) × Epoch (1–6) ANOVA on the P3 peak showed a significant main effect of Type, *F*(1, 31) = 21.87, *p* < .001, η_p_^2^ = .414, indicating that the P3 peak amplitude was larger for pattern than for random triplets (3.74 μV vs. 3.65 μV). This effect suggested a sensitivity to the sequential regularity. The main effect of Epoch, *F*(5, 155) = 1.16, ε = .601, *p* = .328, η_p_^2^ = .036, and the Type × Epoch interaction, *F*(5, 155) = 1.04, *p* = .398, η_p_^2^ = .032, were not significant, suggesting that the overall P3 peak amplitude and its difference between pattern and random triplets did not change as a function of practice (see Fig. 7a). The main effect of Locking, *F*(1, 31) = 3.33, *p* = .078, η_p_^2^ = .097, and the Locking × Epoch interaction, *F*(5, 155) = 0.57, ε = .622, *p* = .641, η_p_^2^ = .018, were not significant, indicating that differences between stimulus-locked and response-locked averages in the P3 peak amplitude could not be reliably detected. More importantly, the Locking × Type, *F*(1, 31) = 0.54, *p* = .469, η_p_^2^ = .017, and the Locking × Type × Epoch, *F*(5, 155) = 1.71, *p* = .136, η_p_^2^ = .136, interactions were not significant either, indicating that the critical experimental effects were comparable between stimulus-locked and response-locked averages (see Fig. 3).

**Fig. 6:**
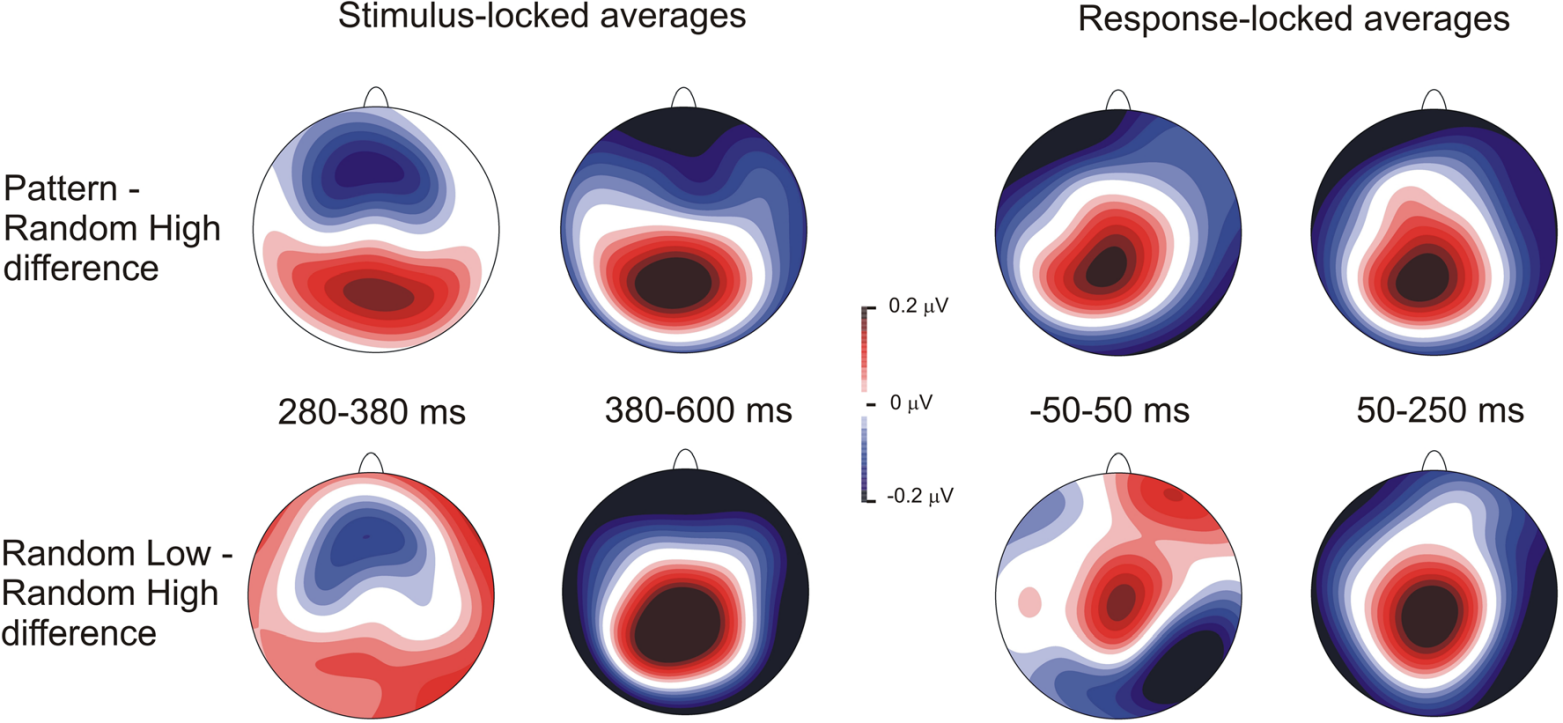
Scalp topographies (amplitude distributions) across all triplet types. Left panel shows the scalp topography of stimulus-locked ERP differences for pattern minus random high-probability triplets (top row) and for random low-probability minus random-high probability triplets (bottom row) in the time windows of the P3 peak (280–380 ms) and the late P3 (380–600 ms), averaged across all epochs. Right panel shows the scalp topography of response-locked ERP differences for pattern minus random high-probability triplets (top row) and for random low-probability minus random-high probability triplets (bottom row) in the time windows of the P3 peak (−50–50 ms) and the late P3 (50–250 ms), averaged across all epochs

**Fig. 7:**
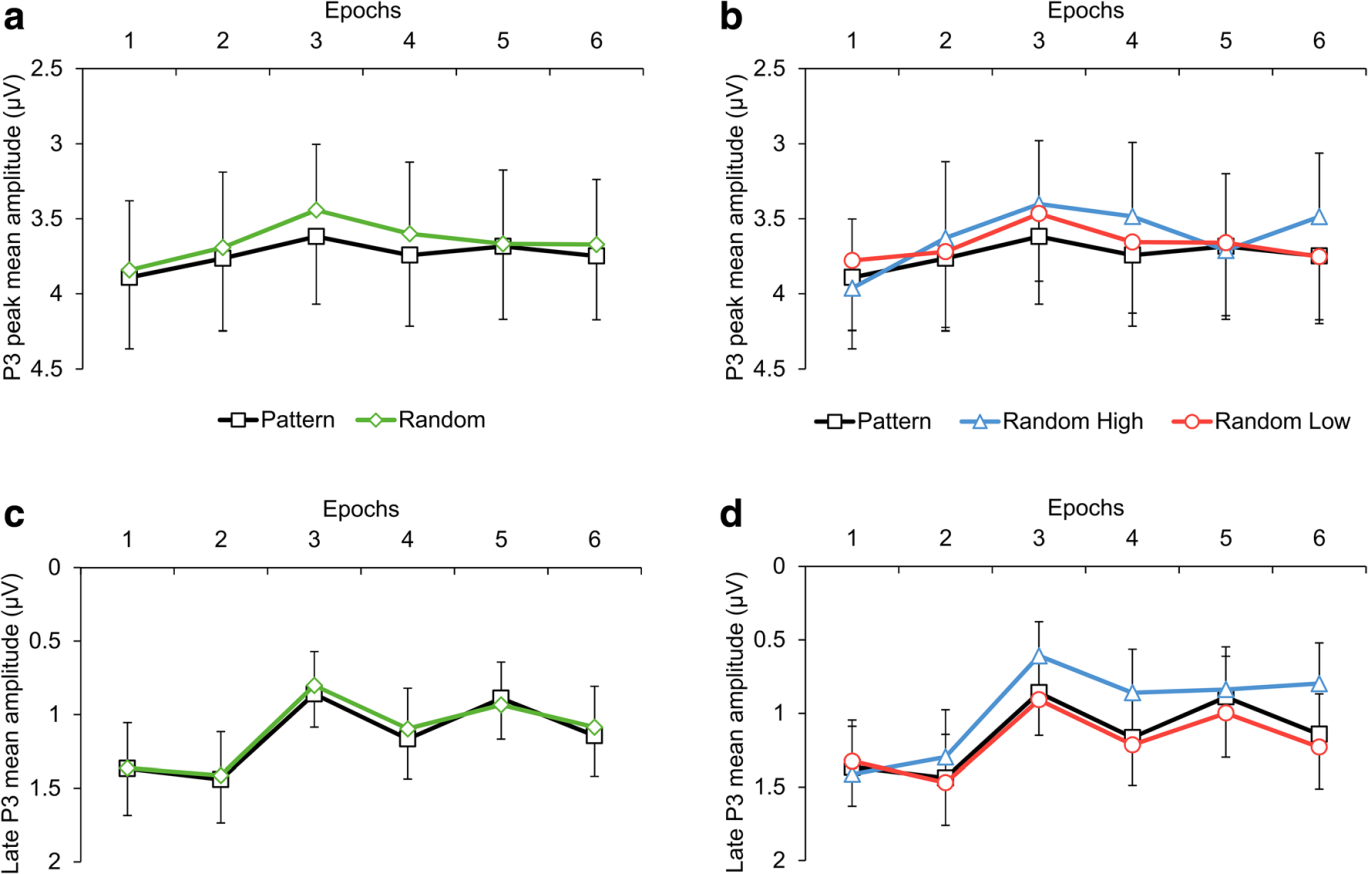
P3 mean amplitude results suggesting sensitivity to the probability structure of the task. Group-average P3 peak (**a, b**) and late P3 (**c, d**) mean amplitudes as a function of epoch (1–6) and triplet type (**a, c**: pattern vs. random triplets; **b, d**: pattern and random high-probability triplets and random low-probability triplets) averaged across stimulus-locked and response-locked segments are presented. Error bars denote standard error of mean

The Locking × Type × Epoch ANOVA contrasting pattern high-probability, random high-probability, and random low-probability triplets on the P3 peak showed a significant main effect of Type, *F*(2, 62) = 5.40, ε = .672, *p* = .017, η_p_^2^ = .148, indicating that the P3 peak amplitude was larger for pattern high-probability than for random high-probability (3.74 μV vs. 3.61 μV, *p* = .002) and random low-probability triplets (3.74 μV vs. 3.67 μV, *p* = .009); however, the random triplets with high and low probability did not differ from one another (3.61 μV vs. 3.67 μV, *p* = .242). This pattern of effects failed to support sensitivity to the second-order transitional probabilities but corroborated sensitivity to the sequential regularity, as only the discrimination between random and pattern triplets became evident. The main effect of Epoch, *F*(5, 155) = 1.40, ε = .646, *p* = .246, η_p_^2^ = .043, and the Type × Epoch interaction, *F*(10, 310) = 1.76, ε = .614, *p* = .108, η_p_^2^ = .054, were not significant, suggesting, again, that the overall P3 peak amplitude and its difference between pattern high-probability and random high- and low-probability triplets did not change as a function of practice (see Fig. 7b). The main effect of Locking, *F*(1, 31) = 3.47, *p* = .072, η_p_^2^ = .101, and the Locking × Epoch interaction, *F*(5, 155) = 0.52, ε = .697, *p* = .696, , η_p_^2^ = .016, were not significant. More importantly, the Locking × Type, *F*(2, 62) = 0.75, *p* = .477, η_p_^2^ = .024, and the Locking × Type × Epoch, *F*(10, 310) = 1.05, *p* = .405, η_p_^2^ = .033, interactions were not significant either, indicating that the critical experimental effects were comparable between stimulus-locked and response-locked averages (see Figs. 4, 5, and 6). Treating the three triplet types separately, the one-way ANOVAs, with Epoch as a within-subjects factor, did not yield any significant effect (pattern high-probability triplets: *F*(5, 155) = 0.78, ε = .606, *p* = .511, η_p_^2^ = .024; random high-probability triplets: *F*(5, 155) = 2.04, *p* = .076, η_p_^2^ = .062; random low-probability triplets: *F*(5, 155) = 1.12, ε = .657, *p* = .348, η_p_^2^ = .035) indicating that no change in the P3 peak amplitude was detectable over the task.

#### Late P3

The Locking (stimulus-locked vs. response-locked averages) × Type (pattern vs. random triplets) × Epoch (1–6) ANOVA on the late P3 revealed only one significant result, the main effect of Epoch, *F*(5, 155) = 5.74, ε = .533, *p* = .002, η_p_^2^ = .156, indicating that the mean amplitude of the late P3 varied during the task, irrespective both of the triplet type and whether it was a stimulus-locked or response-locked component (see below the nonsignificant Locking × Epoch interaction and Fig. 7c). To detail, the late P3 was lower in epoch_3_ [0.83 μV] than in epochs_1,2,4,6_, all *p*s ≤ .020; it was lower in epoch_5_ [0.91 μV] than in epochs_1,2_, all *p*s ≤ .014; and it was lower in epoch_4_ than in epoch_2_ [1.13 μV vs. 1.43 μV, *p* = .033]; overall, a rough decrease was found in the mean amplitude of the late P3 after epoch_2_. The nonsignificant main effect of Type, *F*(1, 31) = 2.03, *p* = .164, η_p_^2^ = .062, and Type × Epoch interaction, *F*(5, 155) = 0.61, *p* = .690, η_p_^2^ = .019, suggested the lack of differentiating the random and pattern elements of the sequential regularity at the level of the late P3 (see Fig. 7c). As in the case of the P3 peak, the main effect of Locking, *F*(1, 31) = 2.18, *p* = .150, η_p_^2^ = .066, and the Locking × Epoch interaction, *F*(5, 155) = 1.72, ε = .741, *p* = .155, η_p_^2^ = .053, were not significant. More importantly, the Locking × Type, *F*(1, 31) = 0.79, *p* = .381, η_p_^2^ = .025, and the Locking × Type × Epoch, *F*(5, 155) = 1.37, *p* = .237, η_p_^2^ = .042, interactions were not significant either (see Fig. 3).

The Locking × Type × Epoch ANOVA contrasting pattern high-probability, random high-probability, and low-probability triplets on the late P3 showed significant main effects of Type, *F*(2, 62) = 17.37, ε = .687, *p* < .001, η_p_^2^ = .359, and Epoch, *F*(5, 155) = 5.90, ε = .542, *p* = .002, η_p_^2^ = .160. These effects were qualified by the significant Type × Epoch interaction, *F*(10, 310) = 2.56, ε = .597, *p* = .021, η_p_^2^ = .076, which suggested the acquisition of second-order transitional probabilities (see Fig. 7d). Pair-wise comparisons showed that the mean amplitude of the late P3 was lower for random than for pattern high-probability triplets in epoch_3_ (0.61 μV vs. 0.86 μV, *p* = .015), epoch_4_ (0.86 μV vs. 1.17 μV, *p* = .003), and epoch_6_ (0.80 μV vs. 1.14 μV, *p* < .001); similarly, it was also lower than for random low-probability triplets in the same epochs (epoch_3_: 0.61 μV vs. 0.91 μV, *p* = .023; epoch_4_: 0.86 μV vs. 1.22 μV, *p* = .005; epoch_6_: 0.80 μV vs. 1.23 μV, *p* < .001). Meanwhile, no significant difference was found between pattern high-probability and random low-probability triplets in any of the epochs (all *p*s ≥ .096). The difference between random and pattern high-probability triplets increased to some extent in the second half of the task (it was larger in absolute value in epoch_6_ [−0.35 μV] than in epochs_1,5_, all *p*s ≤ .011; it was larger in epoch_4_ [−0.31 μV] than in epochs_1,5_, all *p*s ≤ .043). A comparable trend was observed for the difference between random low-probability and high-probability triplets (the late P3 amplitude difference was larger in epoch_6_ [0.43 μV] than in epochs_1,5_, all *p*s ≤ .018; it was larger in epoch_4_ than in epoch_1_ [0.36 μV vs. −0.09 μV, *p* = .011]). In relation to time-on-task effects, pair-wise comparisons also showed that there was a decrease in the late P3 amplitude for random high-probability triplets after epoch_2_ (the late P3 was larger in epoch_1_ [1.41 μV] and epoch_2_ [1.30 μV] than in the remaining epochs, all *p*s ≤ .025), while this decrease was attenuated for pattern high-probability and random low-probability triplets (for pattern high-probability triplets, the late P3 was lower in epoch_3_ [0.86 μV] than in epochs_1,2,4,6_, all *p*s ≤ .022; and, similarly, it was lower in epoch_5_ [0.89 μV] than in epochs_1,2,4,6_, all *p*s ≤ .023; further, it was lower in epoch_4_ than in epoch_2_ [1.17 μV vs. 1.44 μV, *p* = .042]; for random low-probability triplets, the late P3 was lower in epoch_3_ [0.91 μV] than in epochs_1,2,4,6_, all *p*s ≤ .042; and it was lower in epoch_5_ [1.00 μV] than in epochs_1,2_, all *p*s ≤ .039). Again, the main effect of Locking, *F*(1, 31) = 1.91, *p* = .177, η_p_^2^ = .058, and the Locking × Epoch interaction, *F*(5, 155) = 1.68, ε = .783, *p* = .160, η_p_^2^ = .051, were not significant. More importantly, the Locking × Type, *F*(2, 62) = 0.79, *p* = .457, η_p_^2^ = .025, and the Locking × Type × Epoch, *F*(10, 310) = 0.87, *p* = .562, η_p_^2^ = .027, interactions were not significant either (see Figs. 4, 5, and 6).

## Discussion

### Summary of results

This study investigated the ERP correlates of implicitly acquiring second-order nonadjacent transitional probabilities from a sequence of stimuli that shared identical visual characteristics at the surface level. Due to the deep structure of the sequence, sensitivity to a sequential regularity as well as to complex transitional probabilities could be measured. Behavioral results indicated the rapid acquisition of the sequential regularity and that of the transitional probabilities. Namely, on average, participants responded faster to the pattern than to the random stimuli of the alternating sequence. At the level of complex transitional probabilities, their RTs indicated sensitivity to stimulus probability as they responded faster to more than to less probable short-range relations (i.e., triplets) among the random stimuli. Notably, RTs to random high-probability triplets were even faster than to pattern high-probability triplets that were part of the regular sequence. In regard to the ERP correlates, the peak and late phase of the P3 component showed a dissociation regarding their sensitivity to the different regularities. While sensitivity to the sequential regularity was reflected by the P3 peak, sensitivity to the transitional probabilities was reflected by the late P3. The P3 peak amplitude over the centroparietal electrodes was overall larger for pattern than for random stimuli in both stimulus-locked and response-locked averages, which contrasts with our assumption. At the level of complex transitional probabilities, in line with our assumption, the amplitude of the late P3 was larger for less probable than for more probable random triplets. However, it was also larger for triplets that were parts of the sequence (pattern high-probability) than for random triplets with the same probability characteristics (random high-probability). These differences were pronounced in the second half of the task and were not solely related either to stimulus-locked or response-locked averages. In terms of RTs and P3 amplitudes, the processing trajectory of the random high-probability triplets seemed to incorporate that of the pattern and random low-probability triplets, which suggests sensitivity to both the sequential regularity and the transitional probability structure. In sum, considering the main aim of the study, we found evidence for the sensitivity to second-order nonadjacent transitional probabilities at the level of both behavioral and ERP correlates in an active experimental setting where no explicit information was provided or acquired about the sequence underlying the stimulus stream.

### Interpretation of results^1^

In this experiment, all stimuli appeared with the same physical features and were task-relevant targets requiring key presses. Participants did not know that they were in a learning situation and they were not aware of the underlying probability structure of the stimulus stream. (However, some consciously accessible knowledge might have been gained, reflected by the generation performance differing between the inclusion and exclusion condition in the posttask period.) Therefore, although an implicit, incidental, and nonconscious form of acquisition was tested, RT changes and amplitude modulations of the P3 component were found as a function of different regularities.

During the implicit acquisition process, regarding the RT results, the sensitivity to the complex transitional probability structure seemed to emerge first followed by the sensitivity to the sequential regularity. Particularly, the faster responding to the pattern than to the random stimuli of the alternating sequence could be a by-product of differentiating the more probable random and pattern triplets from the less probable random ones. Since the largest RT difference across the triplet types was observed between random high-probability and low-probability triplets (see Fig. 2b), the differentiation between short-range relations based on probability could have played a central role in supporting the extraction of the different types of regularities.

Regarding the ERP results, the acquisition of complex transitional probabilities was suggested by the amplitude of the late P3 being larger for less probable than for more probable random triplets. In the time window where the late P3 was identified, the P3 component returned to the baseline after its peak, and this return seemed to be the earliest for random high-probability triplets, yielding reduced amplitudes. This ERP effect on the late P3 amplitude is in line with and related to the fastest responses recorded to the random high-probability triplets. This, at least at the descriptive level, could also explain the lack of sensitivity to the sequential regularity on the late P3, because when the random high-probability and low-probability triplets were treated together as one category and were contrasted with the pattern triplets, the difference between pattern and random high-probability triplets became covert. In this sense, the observed modulations of the RTs and the late P3 amplitudes might be grounded in the processing of random high-probability triplets.

Meanwhile, the ERP effect on the P3 peak amplitude suggested sensitivity only to the sequential regularity, which, at first sight, was not in agreement with the RT and late P3 results and their interpretation. Although the random triplet types were not differentiated based on probability (high vs. low) in the time window of the P3 peak, the P3 peak amplitude was reliably larger for pattern than for random high-probability triplets (see Fig. 7b). Therefore, it is conceivable that the pattern vs. random discrimination within the high-probability triplet category supported the general discrimination of pattern and random stimuli (i.e., the sensitivity to the sequential regularity). This is elaborated below.

The process underlying the discrimination of pattern versus random high-probability triplets might be guided by the extraction of slightly different probabilistic relations from the stimulus stream. In the case of random high-probability triplets, both the final and the second trials of the triplet are predictable: While the final trial is a predictable continuation for the first trial, the second trial, which is a pattern trial, could be predicted with 100% certainty from the preceding, nonadjacent pattern trial because of the P–[r–P–r] structure (see Fig. 1b). In the case of pattern high-probability triplets, the second trial is a random one, where each of the four stimuli (spatial directions) could occur with 25% probability. Accordingly, both the second and final trials of random high-probability triplets could be considered as “regular,” yielding a short series of predictable stimuli, while this continued regularity does not hold for pattern triplets. Following this argument, the observed modulation of the P3 peak suggesting sensitivity only to the sequential regularity might originate from extracting the respective probabilistic regularities related to the different high-probability triplets. Thus, the decreased P3 peak and late P3 amplitudes for random high-probability triplets possibly result from the enhanced predictability characterizing this triplet type.

One should note, however, that random high-probability triplets are originally rare in the ASRT task, occurring in 12.5% of all trials, which might imply some methodological constraint on the calculation of individual RT and ERP averages for this triplet type. Relatedly, it is worth considering how the order of pattern and random high-probability triplets (i.e., pattern triplets before or after random high-probability triplets) might influence the baseline predictability and processing of these short-range relations. Nevertheless, the present RT as well as ERP effects on the P3 peak and late P3 overall indicate that sensitivity to multiple probabilistic regularities can be established, which is primarily grounded in the implicit extraction of a second-order transitional probability structure.

### Theoretical accounts

The present P3 findings are partially in contrast with our original assumptions. We assumed to obtain overall larger P3 amplitudes for less probable (low-probability triplets) than for more probable (pattern and random high-probability triplets) stimuli of the sequence, irrespective of the exact phase of the stimulus-locked and response-locked P3 component. In addition, we assumed that a two-step acquisition process would determine the P3 amplitude modulations, as sensitivity to the sequential regularity would be observed first (P3 peak) and sensitivity to the transitional probabilities would be observed later in time (late P3). To potentially explain the findings that are more complex than previously assumed, we should consider the current explanatory accounts proposed for the functional significance of the P3 (see also Verleger & Śmigasiewicz, 2016).

The effects on the P3 peak and the late P3 regarding both regularities were equally distinct in stimulus-locked as in response-locked averages. This confirms the notion that the P3 indicates a link between stimulus evaluation and response selection or the process of mapping a task-relevant stimulus onto an appropriate response (e.g., Verleger, 1997; Verleger et al., 2005; Verleger, Schroll, & Hamker, 2013). The concept of stimulus–response link (S–R link), which has been used successfully to model the processing in various oddball tasks, suggests that S–R links established with practice are (re)activated for initiating the correct response during task solving, and the P3 reflects the amount of this reactivation process (Verleger, Grauhan, & Śmigasiewicz, 2016; Verleger, Hamann, Asanowicz, & Śmigasiewicz, 2015; Verleger, Metzner, Ouyang, Śmigasiewicz, & Zhou, 2014; Verleger, Siller, Ouyang, & Śmigasiewicz, 2017; Verleger & Śmigasiewicz, 2016). At the surface level, there are four types of S–R links in the ASRT task (i.e., left-pointing arrow–left response key, etc.), and the four arrow directions (stimulus types) appears with equal probability. However, responding varies according to underlying probability structure, which probably changes the basic S–R links (cf. RT effects). In this respect, after the complex transitional probability structure of the task has been implicitly acquired, responding to low-probability triplets would require S–R link reactivations yielding larger P3 amplitudes, since these links has become infrequently used. Thus, this concept could account for the differentiation of random triplets based on probability in terms of the late P3 amplitudes but could not clearly deal with the ERP effects on the P3 peak and the comparable late P3 amplitudes for pattern and random low-probability triplets. Meanwhile, according to this concept, the reduced S–R link reactivations due to their already frequent use might underlie the P3 effects observed for random high-probability triplets. Overall, this perspective seems to be helpful in explaining the obtained effects, but a subset of the results could be not easily integrated into it.

In line with the S-R link conception, in the sequential sampling framework, the P3 has been considered to indicate the accumulation-to-bound dynamics in decision making, meaning that the P3 possibly tracks the process of decision formation, from sensory encoding until motor preparation (Kelly & O’Connell, 2015; O’Connell et al., 2012; Twomey et al., 2015), rather than adaptation after the decision (Nieuwenhuis et al., 2005). This concept might account for the late P3 amplitude differences within random triplets and the overall reduced P3 amplitudes for random high-probability triplets, because their decision thresholds possibly differ as a function of probability. At the same time, the ERP effect on the P3 peak and the comparable late P3 amplitudes for pattern and random low-probability triplets, again, remains unexplained. Beyond, we should note that potential differences across the triplet types have not been taken into account in the early ascending phase (rise) of the P3, which might have also shown amplitude modulations by processes undergoing before or during decision formation (O’Connell et al., 2012; Verleger et al., 2005). In addition, further studies should manipulate the strength of sensory evidence and/or target difficulty in the ASRT task to directly apply the latter framework in explaining P3 results and to follow an unfolding decision process.

According to the context updating account of the P3, the P3 amplitude could be the marker of the processes by which representations of the varying environment are revised (Donchin, 1981; Donchin & Coles, 1988). In the context of the present experiment, random low-probability triplets could have been considered as unexpected or surprising events, which delivered novel information on the context of the task and could have been related to weaker stimulus–response associations throughout task solving. This possibly resulted in the constant need for reconsidering some of the internal representations of the ongoing stimulus environment, indicated by unchanged and/or larger P3 amplitudes for these triplets. This theory, however, such as the others discussed above, cannot easily account for the lack of difference in the late P3 amplitude between the expected/unsurprising pattern triplets and the random low-probability ones. The P3 peak being larger for pattern than for random triplets does not support this account, either. More importantly, as variations in the late P3 were not exclusively related to the stimulus-locked averages, this theory would rather not, at least fully, explain the present findings.

Variations of the P3 amplitude have been assumed to reflect the employment of increased attentional resources, as well (Polich, 2007; Polich & Criado, 2006). For instance, during the testing phase of auditory statistical learning, in a speeded target-detection task, target syllables in word-final positions elicited the lowest P3 amplitudes compared with word-initial and word-medial positions, indicating that less resources were needed and processing was facilitated for targets in more predictable positions (Batterink, Reber, Neville, et al., 2015; Batterink, Reber, & Paller, 2015). Hence, in the present study, more probable triplets should have elicited lower P3 amplitudes. Although the reduced P3 amplitudes for the random versus pattern high-probability triplets could be interpreted in this framework, it is challenging to explain the comparable P3 peak amplitudes for the random triplet types and the comparable late P3 amplitudes for the pattern and random low-probability triplets. One should take into account that reduced P3 amplitudes could also indicate decision uncertainty and that the resources available for stimulus processing are needed elsewhere during effortful processing (Beauducel, Brocke, & Leue, 2006; Johnson, 1986; Kok, 2001). In this sense, findings on the random high-probability triplets might suggest that this triplet type was somewhat harder to process than the others, but, as described above, its enhanced predictability related with faster processing implies the opposite. In addition, it is not clear how much attentional resources the fully implicit acquisition of a complex transitional probability structure involves (cf. both probabilistic and deterministic information in Batterink, Reber, & Paller, 2015). Overall, relating the observed findings to the allocation of attentional resources is not entirely helpful.

The context closure hypothesis should also be considered, since it has appeared as an explanatory account in certain ERP studies on the acquisition of predictive relations (e.g., Daltrozzo et al., 2017). This hypothesis posits that the P3 is elicited when expectancies are fulfilled after associations have been learned across successive elements in repetitive, highly structured tasks (Verleger, 1988). More formally, the P3 is elicited when the given stimulus is expected to close the perceptual epoch or chunk. If such a chunking process had underpinned the observed effects in the present study, the P3 amplitude would have been the largest for the last relevant closing event of a three-element-long trial sequence (i.e., for pattern as well as random high-probability triplets), especially in the final phase of the task. In line with this assumption, larger P3 peak amplitudes for pattern than for random triplets were found, which also corresponds to previous studies showing larger P3 amplitudes for stimuli with high predictive value (Baldwin & Kutas, 1997; Daltrozzo et al., 2017; Fogelson & Fernandez-del-Olmo, 2013; Jost et al., 2015; Rose et al., 2001; Rüsseler et al., 2018; Stadler et al., 2006). However, opposite to what this framework suggests, reduced P3 amplitudes were found for random high-probability triplets and late P3 amplitudes were comparable between pattern and random low-probability triplets.

Although chunk learning has been found to be an important contributor to implicit statistical learning (e.g., Batterink, 2017; Perruchet & Pacton, 2006), it has recently been suggested that both rule-based statistical computation and chunk learning operate during this learning form (Christiansen, 2018; Fu, Sun, Dienes, & Fu, 2018). This would be plausible for the ASRT task, as well. However, it is likely that not only the abstraction of three-element-long chunks takes place during acquisition but also shorter-ranging and longer-ranging chunks organized upon predictability are processed (e.g., Kóbor et al., 2018; Meyniel, Maheu, & Dehaene, 2016; Soetens & Notebaert, 2005), as it was suggested above in regard to the structure of random high-probability triplets. Other examples for long-range chunks are also conceivable: In the present task version, the oscillation of pattern high-probability and random low-probability triplets could have constituted a basic repeating sequence unit in the alternating sequence, which was occasionally violated by the random high-probability triplets. The predictability between every second trial was 100% in the sequential regularity, which, although sequence knowledge remained implicit, could have facilitated stimulus evaluation and responding for the expected pattern and random low-probability triplets, yielding enhanced late P3 amplitudes (cf. Baldwin & Kutas, 1997; Fogelson & Fernandez-del-Olmo, 2013). This idea, however, is not plausible for the P3 peak findings showing reduced amplitudes for the random low-probability triplets. Since the boundaries and the expected closing stimuli of the plausible chunks currently seem to be unclear in this task, the context closure hypothesis cannot provide an exhaustive account for the observed P3 findings. Moreover, the perceptual epoch as the central concept of this theory appears to be problematic not only in relation to the structure of the task but also because the P3 findings are related to both the stimulus-locked and response-locked averages, thus, the involved processes are not solely perceptual.

Taken together, none of the discussed concepts about the P3 in their present forms could fully account for the observed P3 findings. The notion of S–R link reactivation appears to be partially tenable but only with caveats. Particularly, the late P3 amplitudes being similar between pattern and random low-probability seems to challenge the delineated theories. We proposed above that the alternation of these triplets might be a basic repeating sequence unit, and, therefore, they could be awaited or relevant (cf. Johnson, 1986) but only in a strictly implicit manner. Another study directly enhancing the implicit extraction of the sequential regularity independent from that of the transitional probability structure (cf. Deocampo et al., 2019) might provide some insight on this issue.

### Relating the P3 findings to acquisition processes observed in different ASRT task versions

In the present implicit experimental design, the P3 amplitude modulations might reflect a general process by which stimuli with different statistical-sequential properties are implicitly categorized and consequences of the related behavioral responses are evaluated to maintain (or increase) ongoing performance (cf. Nieuwenhuis et al., 2005; Verleger et al., 2005). The idea that motor representations are continuously revised and strengthened during the online acquisition of second-order transitional probabilities is consistent with those findings on the ASRT task that suggest similar importance of the perceptual and motor components of the implicit learning processes underlying task solving (Hallgató et al., 2013; Nemeth, Hallgato, Janacsek, Sandor, & Londe, 2009). Although mere observation of the probabilistic sequence was also found to be sufficient to acquire second-order transitional probabilities in a modified ASRT task (Song, Howard, & Howard, 2008), this perceptual learning process was vulnerable to task demands. This finding also supports that the integration of motor response representations could have a complementary role in mapping the probabilistic relations of the varying stimulus–response environment.

The differential sensitivity of the P3 component to nonadjacent transitional probabilities has already been observed in the cued ASRT task. We showed that the stimulus-locked P3 amplitude was only sensitive to the gradual acquisition of complex sequential structures (pattern vs. random high-probability triplets) and not to that of the statistical probabilities (random high-probability vs. low-probability triplets), when the predetermined elements of the alternating sequence were explicitly marked by black arrows and the random elements were marked by red ones (Kóbor et al., 2018). However, in that study, the P3 was quantified in a narrow time window, as the mean amplitude between 250 ms and 350 ms at the electrode Pz, which covered mainly the peak of the component. As it is observable in Fig. 5 in that paper, the late phase of the P3 (after approx. 380 ms) also shows some variation between random high-probability and random low-probability triplets, although primarily in the first half of the task. Thus, possibly, it was only the peak of the P3 component that did not show sensitivity to the extraction of statistical probabilities, which is in line with the current findings that sensitivity to the second-order nonadjacent transitional probability structure cannot be reliably captured at the peak of the P3. Meanwhile, it should be noted that the subjective probability of the different triplet types is altered in the cued ASRT task, since explicit knowledge about the sequential regularity (i.e., the direction of the next pattern stimulus) emerges early during task solving (Kóbor et al., 2018; Nemeth, Janacsek, & Fiser, 2013; Simor et al., 2019). This renders the pattern triplets the most predictable ones, modulating the temporal change in RTs and P3 amplitudes for these triplets in a different manner than in the present implicit task version. Nevertheless, our previous and present findings altogether suggest that multiple processes that are responsible for the acquisition of complex statistical properties of a structured stimulus stream can be differentiated using ERPs (cf. Maheu et al., 2019).

The mean RT and the late P3 amplitude also showed some nonlinear variations across the six time bins of the task (indicated by the significant Epoch main effect). This, at least partly, can be explained by reactive inhibition (Brawn, Fenn, Nusbaum, & Margoliash, 2010; Pan & Rickard, 2015). This phenomenon has already been observed in our previous behavioral studies using variations of the ASRT task (Kóbor et al., 2017; Simor et al., 2019; Török, Janacsek, Nagy, Orbán, & Nemeth, 2017), and could originate from our general experimental procedure. Here, after ten and twenty blocks (two and four epochs), respectively, a few-minute-long break was inserted to check the impedance levels of the electrodes. It is possible that mean RTs and late P3 amplitudes decreased in the successive epochs (epoch_3_ and epoch_5_) for pattern and random low-probability triplets because of these somewhat longer rests. However, such change was not observed for random high-probability triplets. They remained basically unchanged in terms of the mean RTs and were related to decreased late P3 amplitudes after the first ten blocks (epoch_1_ and epoch_2_), which might be explained by the assumed enhanced predictability of the stimulus series in these triplets. Still, the diverse processing trajectory of the random high-probability triplets across time bins suggests the processing of both the sequential regularity and the second-order transitional probability structure.

### Conclusions and future directions

We found behavioral and neurophysiological evidence for the implicit acquisition of second-order nonadjacent transitional probabilities embedded in a sequence of visual stimuli during an active experimental setting. The differentiation of the P3 component into its peak and late descending flank promoted the fine-grained analysis of the acquisition process. Particularly, in line with the RT effects, the observed P3 amplitude effects, both in stimulus-locked and response-locked averages, indicate that the extraction of the underlying regularities is primarily based on the implicit distinction of short-range relations differing in transitional probabilities. The results suggest that when the probabilistic relations of the stimulus environment should be implicitly mapped, the P3 reflects a process by which the stimulus–response contingencies are acquired. In sum, the role of predictive processes during implicit memory formation and the automatic extraction of complex temporal sequences crucial in many day-to-day situations are emphasized.

The experimental design is also noteworthy from a methodological point of view. It could enable us to investigate the temporal trajectory of acquiring nonadjacent transitional probabilities in patients with movement disorders such as Huntington’s disease and Parkinson’s disease. In these disorders, impaired acquisition of probabilistic regularities originating from abnormalities in the subcortical structures have been found (Clark, Lum, & Ullman, 2014; De Diego-Balaguer et al., 2008). Yet, administering tasks that require overt behavioral responses could be challenging in these clinical populations, and the putative conclusions drawn from the data, might be less reliable. With the help of ERPs, especially the P3 component, one could gain a better understanding of the intact and altered characteristics of multiple incidental acquisition processes (e.g., Verleger et al., 2013).

## Footnotes

1 We integrated some ideas of one of the reviewers into this section.
